# Supplementary material for: One-step generation of multiple gene knock-outs in the diatom Phaeodactylum tricornutum by DNA-free genome editing
Source: Nat Commun. 2018 Sep 25;9:3924. doi: 10.1038/s41467-018-06378-9 (PMC6156588; doi:10.1038/s41467-018-06378-9)
Supplement: Supplementary file 1 — Supplementary Information [file 41467_2018_6378_MOESM1_ESM.pdf]

One-step generation of multiple gene knock-outs in the diatom *Phaeodactylum tricornutum* by DNA-free genome editing

Serif et al.

ATGGCCACCCCCTCTTTTCGATCAAAGCTTGAAGCTCGAGTCGCCGCAGTCAACTCTCTCTTGTGCGTTGGTCT
AGACCCGCACGAGAAAGAGCTGTTTGCAGACGGATGGGAAGGCGTGCCGGAAGAAAATCGCTGTGACGCGGCCT
TTACCTTTTGCAAAACGTTGGTCGACGCAACATTGCCTTACACGGCCTGCTACAAACCCAATGCTGCCTTTTTC
GAGGCGTTAGGCGATGGAGGGATGGCGGTTCTGCGACGAGTT**TGTCAAAACATAATACCGGATGATGTGCCGAT**
TTTGTTGGATGTCAAGCGCGGCGACAT***TGG***CTCGACCGCTGCGGCCTACGCCGAAGCGTGCTATGGTTTGGGTG
CAGACTGTGTCACGCTTTCACCACTGATGGGATGGGACTCAGTCAGTCCCTTTGTTACAGGTAAGTTGTAGCTA
AAGGATTGGCATCTCCAGCGATTGAATGGATGCCATCTGATACGTTGAATTGACTCCCTCCGACAGAAAAGTAC
GTTCACAAAGGAGCATTTTTGCTGTGCAAAACGTCAAATCCTGGATCCAACGATTTTTTAGCTCTGGGATTACG
TTCAAATGAATGTTTATACGAAAGAATTGCCAAGCTTGTTGGCTCGGAAT***GGG***CTCAGCAGACCGAGAGTTCAT
TGGGACTCGTTGTCGGGGCCACAGATCCAGTGGCCTTGTCCAAAGCGAGAAAGGCTGCAGGCGACGACACCTGG
ATTCTAGCACCCGGCGTTGGTGCTCAAGGTGGAGATCTTCTAGAAGCAGCGCAGGCTGGATTGAATACAAAGGG
GACTTGCATGCTAATTCCCGTGTCTAGGGGTATCAGCAAAGCTACGGACCCAGCGCAGGCTGCAAAAGAATTGC
AGGAGAGGATTCAGAAAGCTCGGGACCAAGTCGTGGCCGCACACATGATGAAAAAGAGTTCAGACGAAGATATT
AAACTCTATCAACGCGAGTTTCTTGAATTTAGTCTGTCTCAAGGTGTTCTCAAATTCGGCTCTTTTGTGCTGAA
AAGCGGCCGCACCTCTCCATATTTTTTCAACGCCGGTCTTTTTGCTTCTGGCGCTGCGTTAAGCAAGCTTGGGA
AAGCCTATGCTTCGACTATCATGTCCTCGGAATTATTGTAAGTGTGCTTTGTGTGTTTTTCTCTGCTGAACGGC
AAAAATTCAAGAGAAGGATGAGTATCCACTTGGTCCGTGTTACCGATCTGCCCCCACGTGAGTGGCAATGAGCA
AATTTTTTTCCAGTGGCCTGACTCTTGAAACAACATAGTCGATGATGACTCCTTTGGTCTTCTTTCACCTAATT
TCTCCGGAAAGATGCCGGTCAACACCAATTCGCTGATTCGAAATTTTCTGAGACTGTGTTTTGATTTAGTTCTA
TGGGACTATCATTGTTGTGAGCAGGCTTACCCAACAAAATTCGTTTTCTTTTTCCTTCCAAAGCTGCTGGGCCC
AACCAAGTCAATTTTGATGTGATTTTTGGTCCTGCATACAAGGGTATTTCTCTAGGTGCTGTCGTTGGAAGCGC
TCTGTATAACGATTTTGAAGTAGATGTCGGTTTTGCGTATGACCGAAAAGAGGCAAAGGATCATGGGGAAGGTG
GTAAATTGGTCGGGACTTCGTTGGAAGGAAAACGAGTTCTGATTGTAGATGACGTAATCACAG***CGG***GAACCGCC
ATTCGTGAGTCGCACACTTTGCTCAACGATGTGGGTGCTTTGCCAGTTGGAGTAGTTATTGCCCTCGATCGAGC
CGAAATTCGCTCTATGGAGGACAAGATTTCCGCTGTTCAAGCAGTCGCACGAGATCTATCTCTTTTGGTCGTGT
CAATTGTCAGTCTTCCTCAACTACAGACATTTCTCGAACGAAGTCCGGACTACGGCGATGAAACGCTGGAAAAA
GTAATTAAGTATCGAAACGAATACGGAGTGTAATGAATAACTTTTTGATCAAACTGACTTAATACTTCGGTTAA
CAACTAAATACTCTGGCGAGGTTTTCCACACAACAGAGACTTTGACGGAA

Supplementary Figure 1. Sequence of the *PtUMPS* gene amplified from wild type *Phaeodactylum* cells. Red letters indicate the positions where a single nucleotide polymorphism was observed between the alleles. Bold, dark blue letters represent the TALEN targeting region. The gUMPS1 (dark blue), gUMPS3 (light blue) as well as gUMPS4 (medium intensity blue) target sites are underlined and their proto-spacer adjacent motives displayed in italics.

F/2 medium + 50µg mL^{-1} Uracil + 100µg mL^{-1} 5-FOA

a

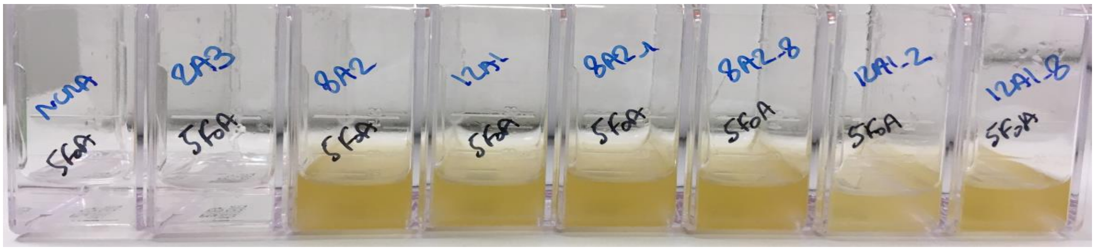

F/2 medium

b

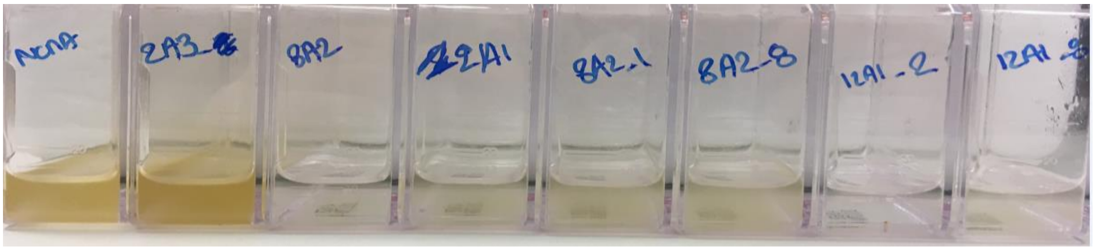

F/2 medium + 50µg mL^{-1} Uracil

c

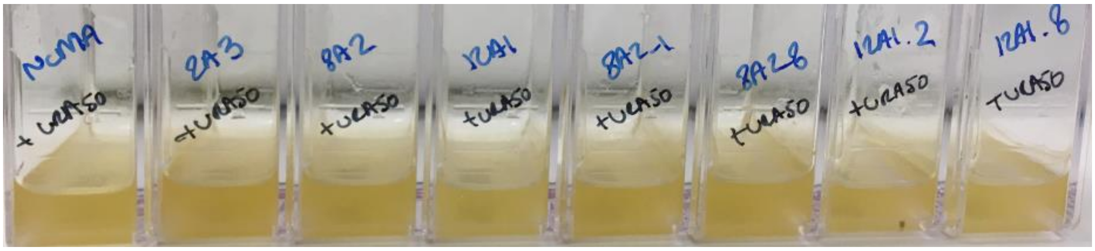

Supplementary Figure 2. Pictures of the TALEN-generated *PtUMPS* knock-out strains after 10 days of culture. Cultures of the parental NCMA strain, the 2A3 control strain transformed with the NAT vector only, two 5-FOA-resistant populations (8A2 and 12A1), and four subclones: 8A2_1, 8A2_8, 12A1_2 and 12A1_8 in (**a**) F/2 medium supplemented with 50 µg mL^{-1} uracil and 100 µg mL^{-1} 5-FOA, (**b**) classical F/2 medium, or (**c**) F/2 supplemented with 50 µg mL^{-1} uracil.

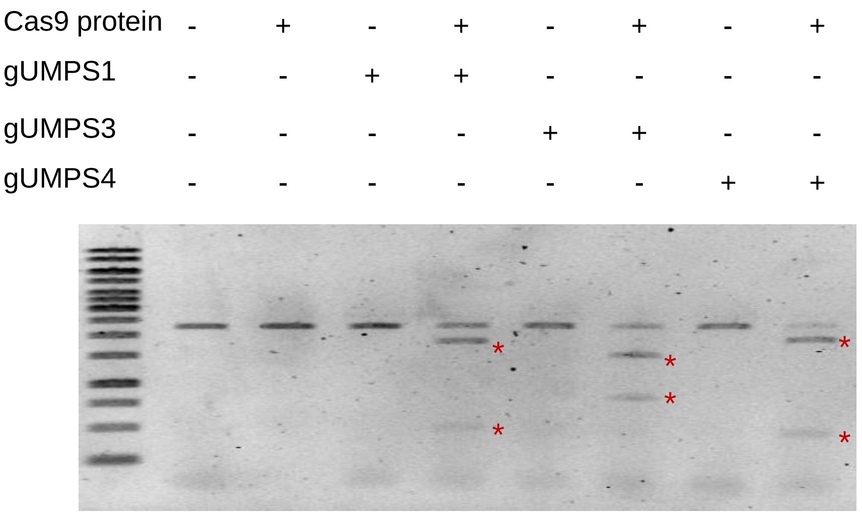

Supplementary Figure 3. CRISPR/Cas9 RNP *in vitro* cleavage assays for three gRNAs targeting *PtUMPS*, gUMPS1, gUMPS3 and gUMPS4. PCR amplicons were incubated with (+) or without (-) sgRNA and Cas9. Red asterisks indicate cleavage products at the expected size.

Cells transformed with the Cas9 and empty U6 vectors

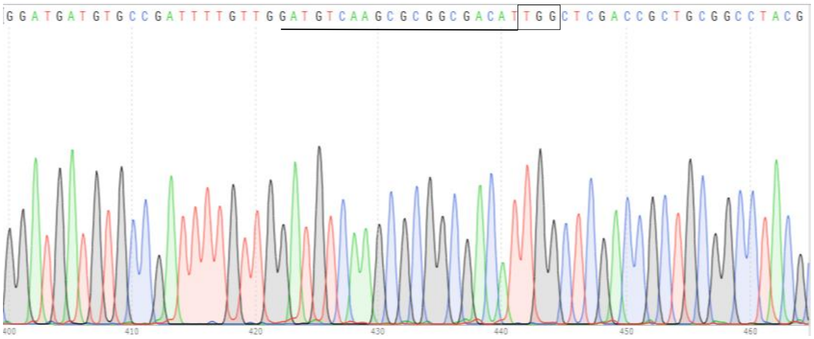

Cells transformed with the Cas9 and **gUMPS1** encoding vectors Clone 1

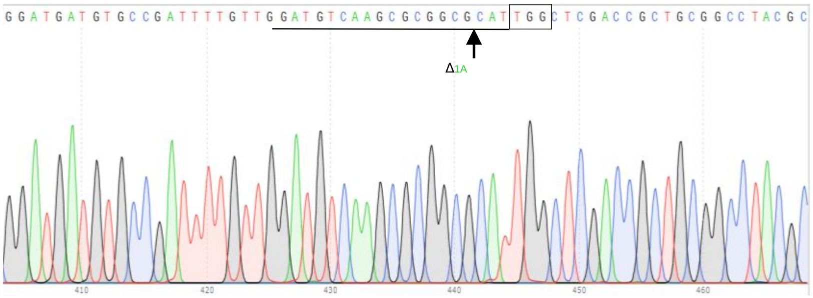

Cells transformed with the Cas9 and **gUMPS1** encoding vectors Clone 2

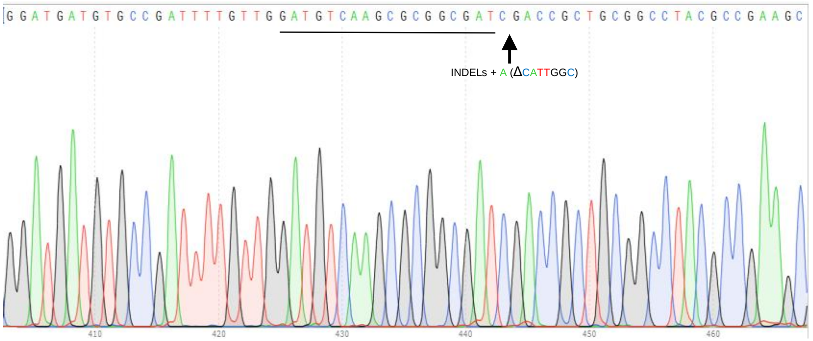

Supplementary Figure 4. Sequencing chromatograms of the gUMPS1 site in wild type cells (top) and in two independent 5-FOA-resistant clones obtained after transformation with the Cas9 and gUMPS1 encoding vectors. The gRNA target site is underlined and the PAM (TGG) sequence boxed.

Cells transformed with the
Cas9 and empty U6 vectors

Cells transformed with the
Cas9 and **gUMPS3**
encoding vectors
Clone 1

Cells transformed with the
Cas9 and **gUMPS3**
encoding vectors
Clone 2

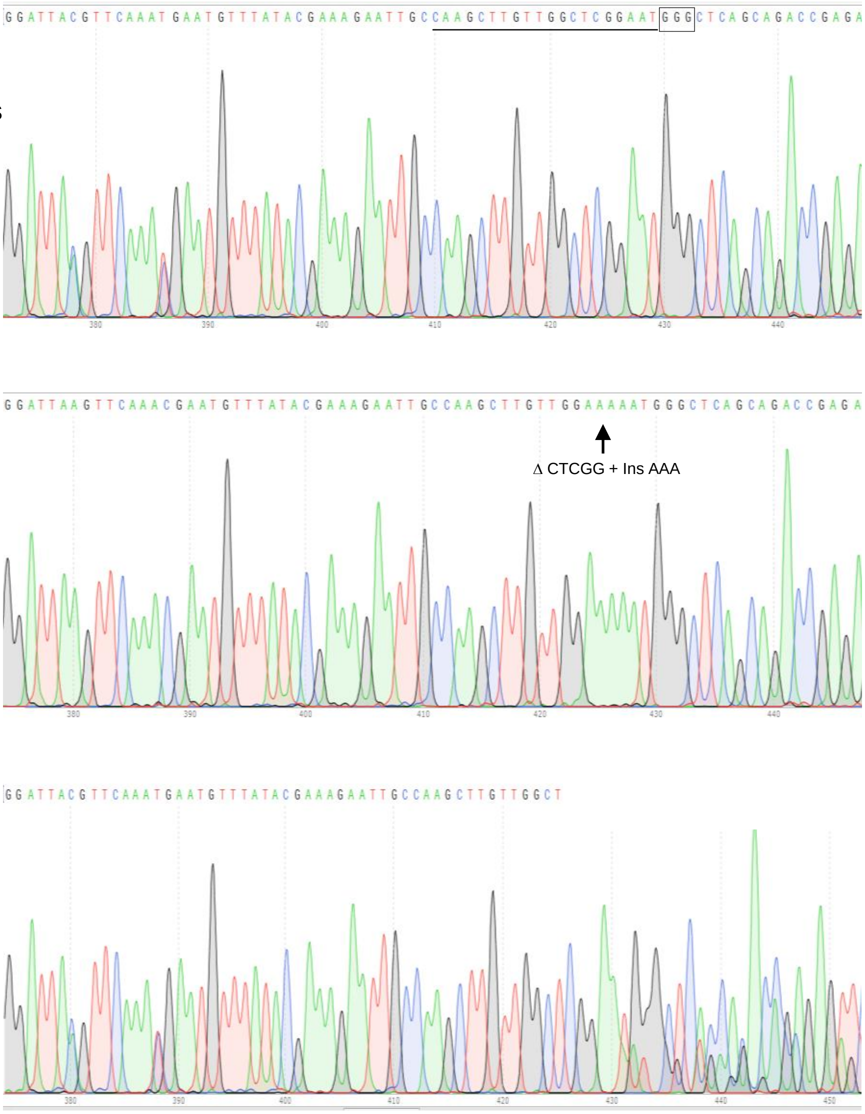

Supplementary Figure 5. Sequencing chromatograms of the gUMPS3 site in wild type cells (top) and in two independent 5-FOA-resistant clones obtained after transformation with the Cas9 and gUMPS3 encoding vectors. The gRNA target site is underlined and the PAM sequence (GGG) boxed.

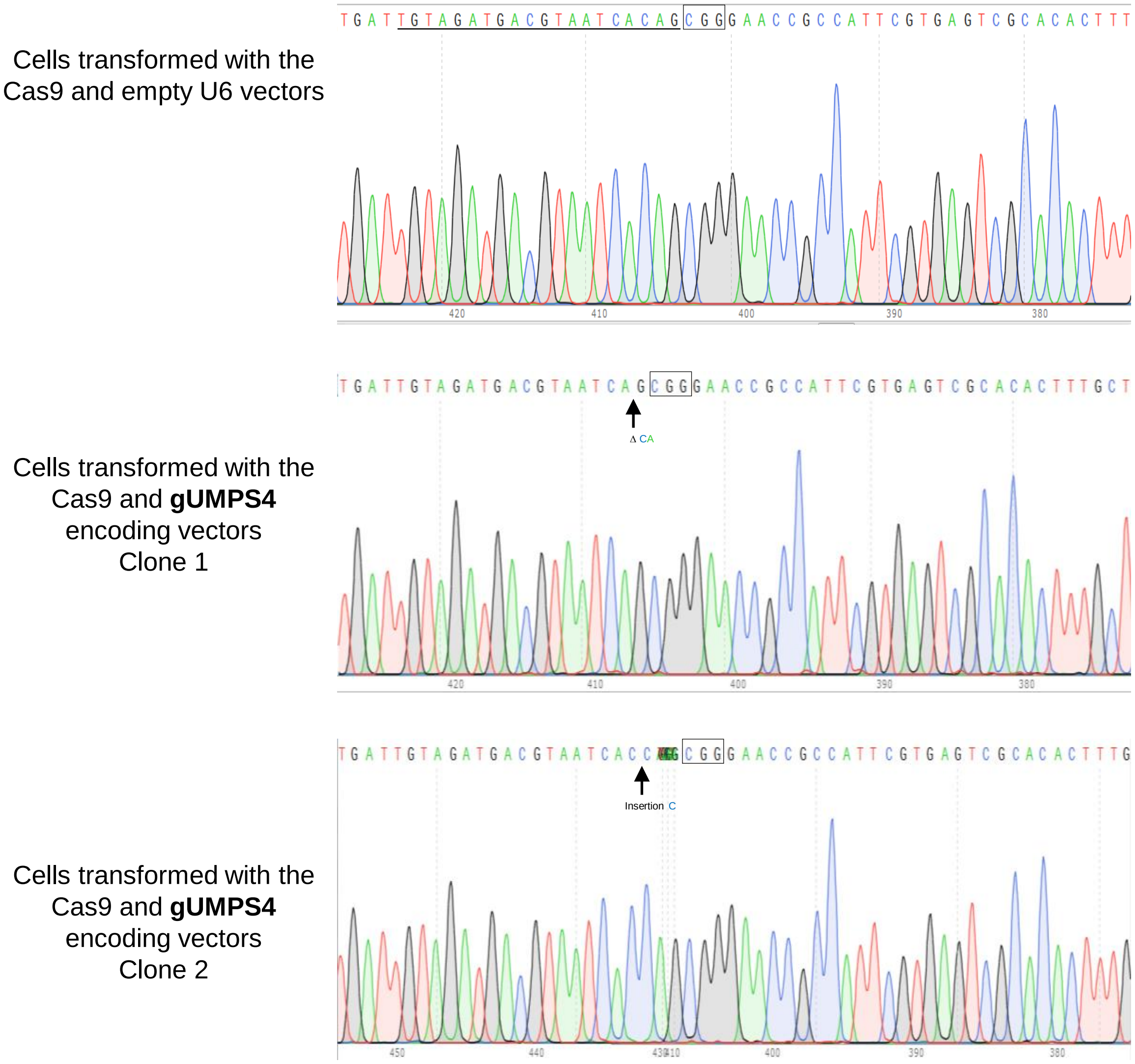

Supplementary Figure 6. Sequencing chromatograms of the gUMPS4 site in wild type cells (top) and in two independent 5-FOA-resistant clones obtained after transformation with the Cas9 and gUMPS4 encoding vectors. The gRNA target site is underlined and the PAM sequence (CGG) indicated

a

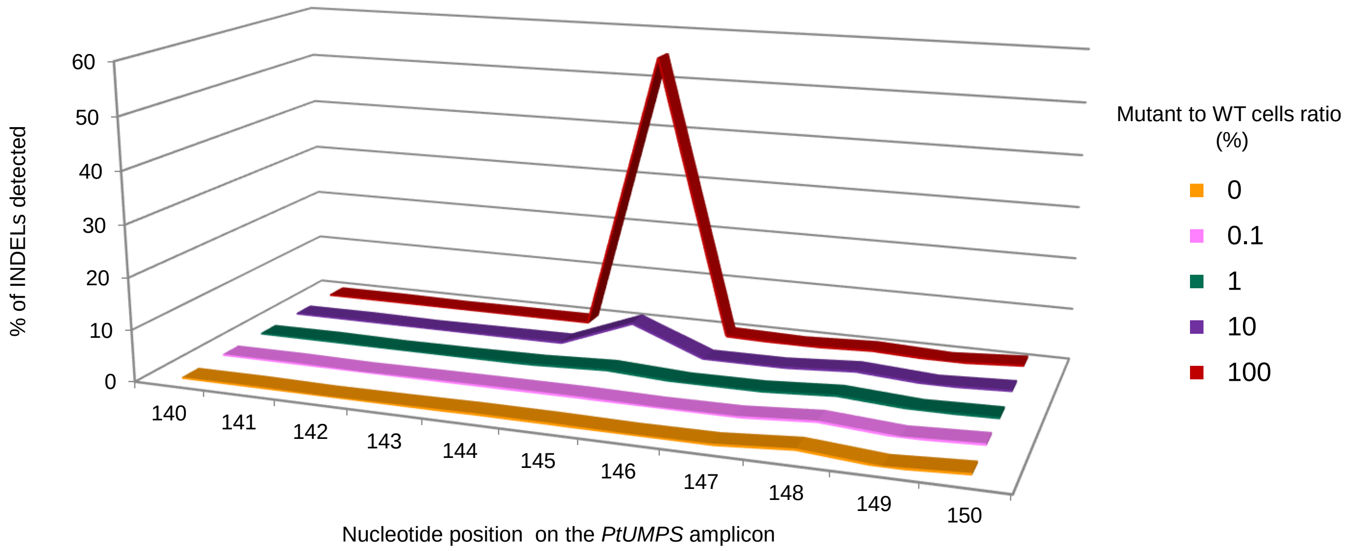

b

Condition	% of expected INDELs at position 145	Total number of sequences	Number of sequences with INDELs	% of INDELs detected		% of detected INDELs longer than 1 nucleotide	
	Position 145	Position 145	Position 145	Position 145	+/- 5bp surrounding position 145	Position 145	+/- 5bp surrounding position 145
**P100**	50	47 349	26 240	55,4	0,24	0	0,01
**P10**	5	31 075	1 778	5,72	0,26	0,01	0,01
**P1**	0,5	66 066	606	0,87	0,24	0,04	0,01
**P0,1**	0,05	25 116	67	0,26	0,27	0,,02	0,01
**P0**	0	30 600	89	0,29	0,27	0,04	0,02
RNP UMPS1 0µg	NA	71 872	224	0,31	0,29	0,03	0,01
RNP UMPS1 2µg	NA	20 971	44	0,21	0,32	0,02	0,01
RNP UMPS1 4µg	NA	13 568	46	0,34	0,30	0,05	0,01
RNP UMPS1 8µg	NA	25 646	57	0,22	0,24	0,04	0,02

Supplementary Figure 7. Deep-sequencing of *PtUMPS* amplicons derived from cells bombarded with different amounts of RNP gUMPS1 (0-2-4-8 µg). **(a)** Mixtures of a monoallelic mutant strain carrying a 1nt deletion at the gUMPS1 target site (position 145) with WT cells to get cell-to-cell ratios of 100% (Red), 10% (Purple), 1% (Green), 0,1% (Pink) and 0% (Orange) were used as positive controls. **(b)** Overall INDEL frequencies measured by targeted deep sequencing for RNP gUMPS1 (Blue) and the samples corresponding to the control range. The percentage of mutagenic event was calculated at position 145 (3nt upstream of the PAM, expected cleavage site) and at +/- 5 bases surrounding this position. The frequency of total INDELs (including 1 nucleotide INDELs) was calculated. The background noise level was around 0,3% and corresponded to PCR and sequencing errors. The frequency of INDELs larger than 1 base was also included in this table, with a background noise of 0,02%. We detected no TM induced in the samples bombarded with RNP Cas9-gUMPS1 compared to the condition with Cas9 alone. **NA** : Not Applicable.

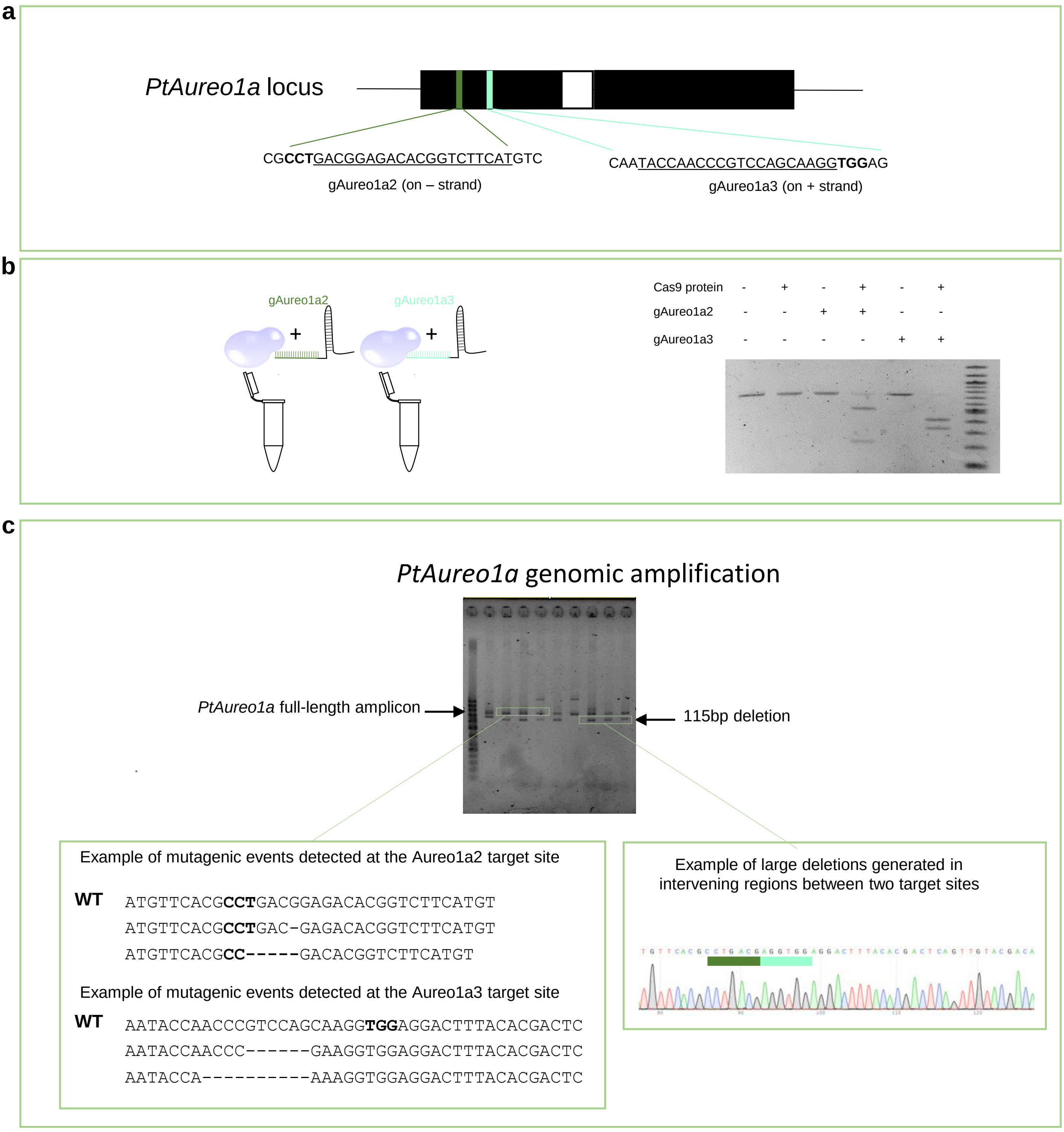

Supplementary Figure 8. (a) Schematic representation of the *PtAureo1a* locus. Exons are represented as black boxes, introns as white boxes. The target sequences for gAureo1a2 (- strand) and gAureo1a3 (+ strand) are underlined and the associated PAMs are shown in bold font. **(b)** CRISPR/Cas9 RNP *in vitro* digestion assays for gAureo1a2 and gAureo1a3. PCR amplicons of *PtAureo1a* were incubated with (+) or without (-) sgRNA and Cas9. **(c)** Amplification of the *PtAureo1a* locus from 9 of the 5-FOA-resistant clones that appeared after transformation with a mixture of RNP complexes targeting *PtUMPS* and *PtAureo1a*. Lane 1: 1kb DNA ladder; lanes 2-10: amplicons from the 5-FOA^{R} clones. Inset show examples of mutagenic events, for either the higher or lower band detected.

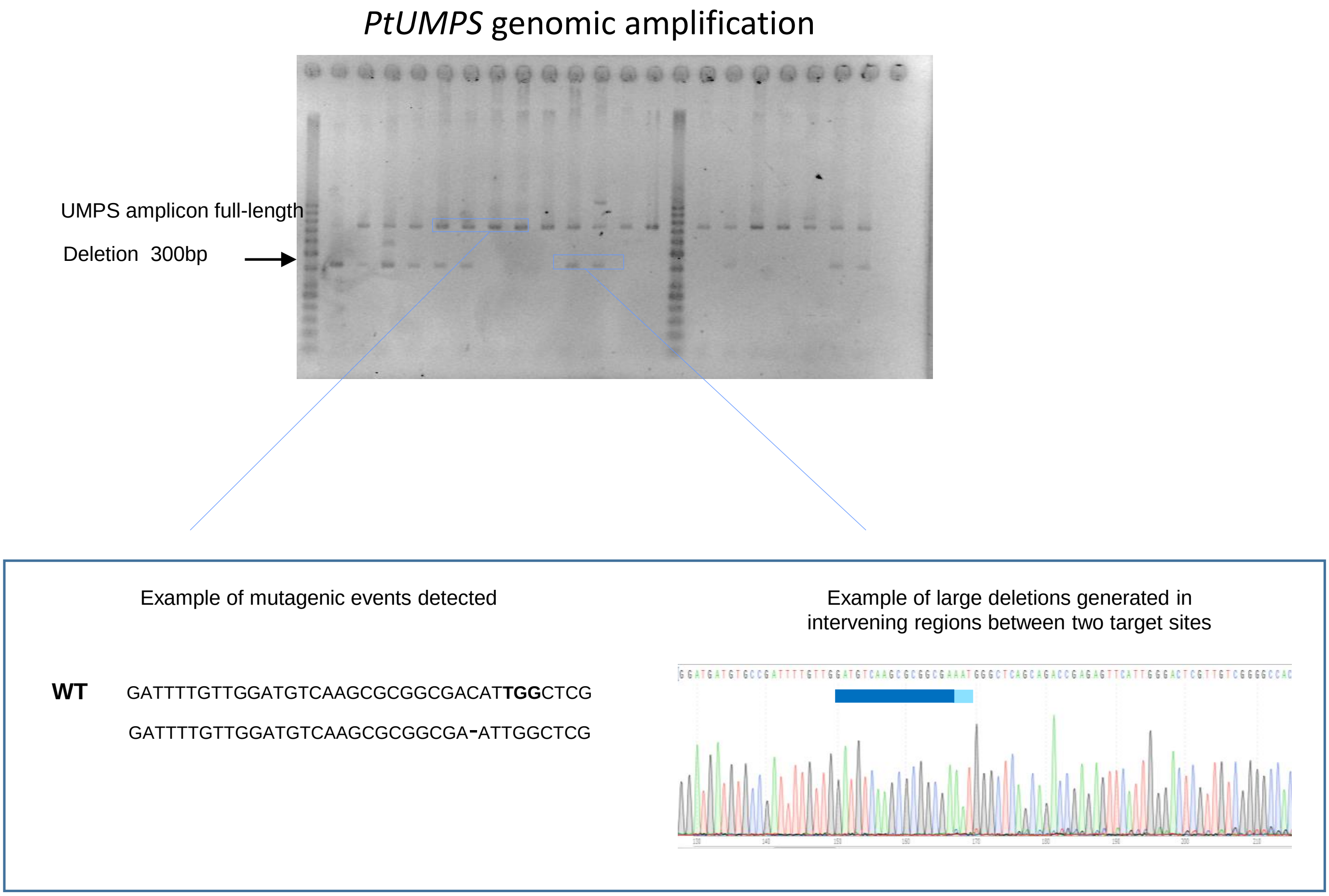

Supplemental Figure 9. Amplification of the *PtUMPS* loci from 19 5-FOA-resistant clones that appeared after transformation with a mixture of RNP complexes targeting *PtUMPS* and *PtAureo1a*. Classical, none allele-specific PCRs were performed. Lane 1: 1kb DNA ladder; lanes 2-13: amplicon from twelve of the 5-FOA^R clones; lane 14: amplicon from WT; lane 15: 1kb DNA ladder; lanes 16-22: amplicons from seven of the 5-FOA^R clones. Inset shows example of mutagenic events, for either the higher or lower band detected.

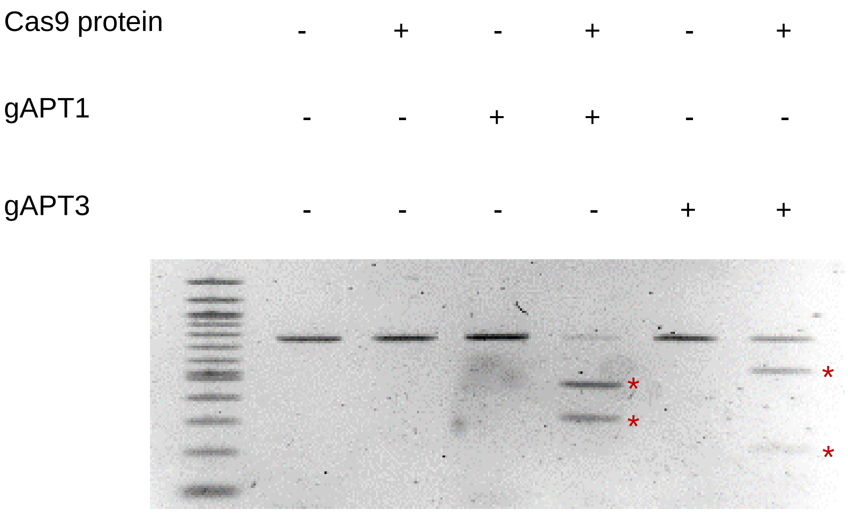

Supplementary Figure 10. CRISPR/Cas9 RNP *in vitro* digestion assays for two gRNAs targeting *PtAPT*, gAPT1 and gAPT3. PCR amplicons were incubated with (+) or without (-) sgRNA and Cas9. Red asterisks indicate cleavage products at the expected size.

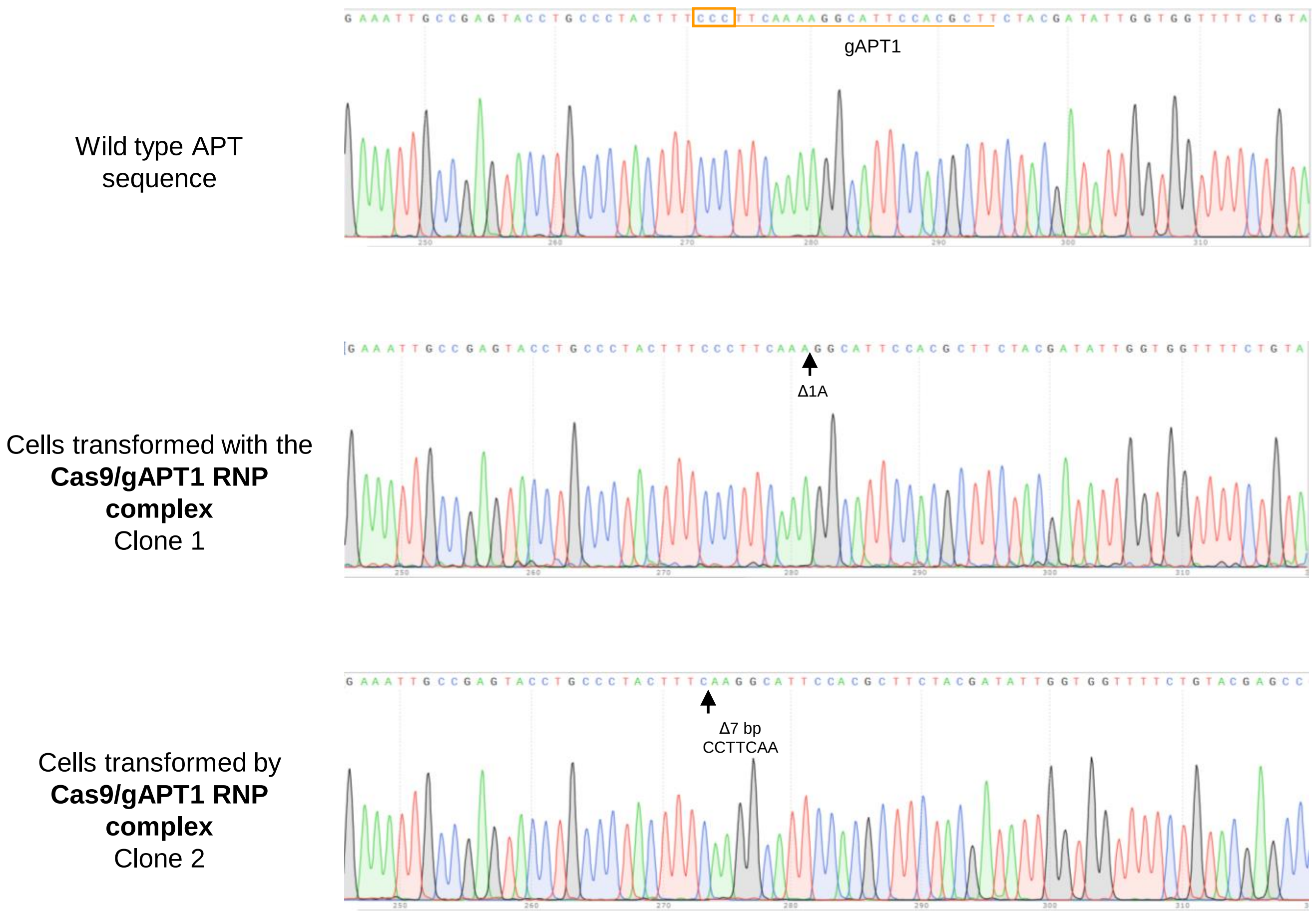

Supplementary Figure 11. Sequencing chromatograms of the gAPT1 site in wild type cells (top) and in two independent 2-FA-resistant clones obtained after transformation with the Cas9/gAPT1 RNP complex. The gRNA target site, situated on the reverse strand, is underlined and the PAM sequence (- strain: GGG) boxed.

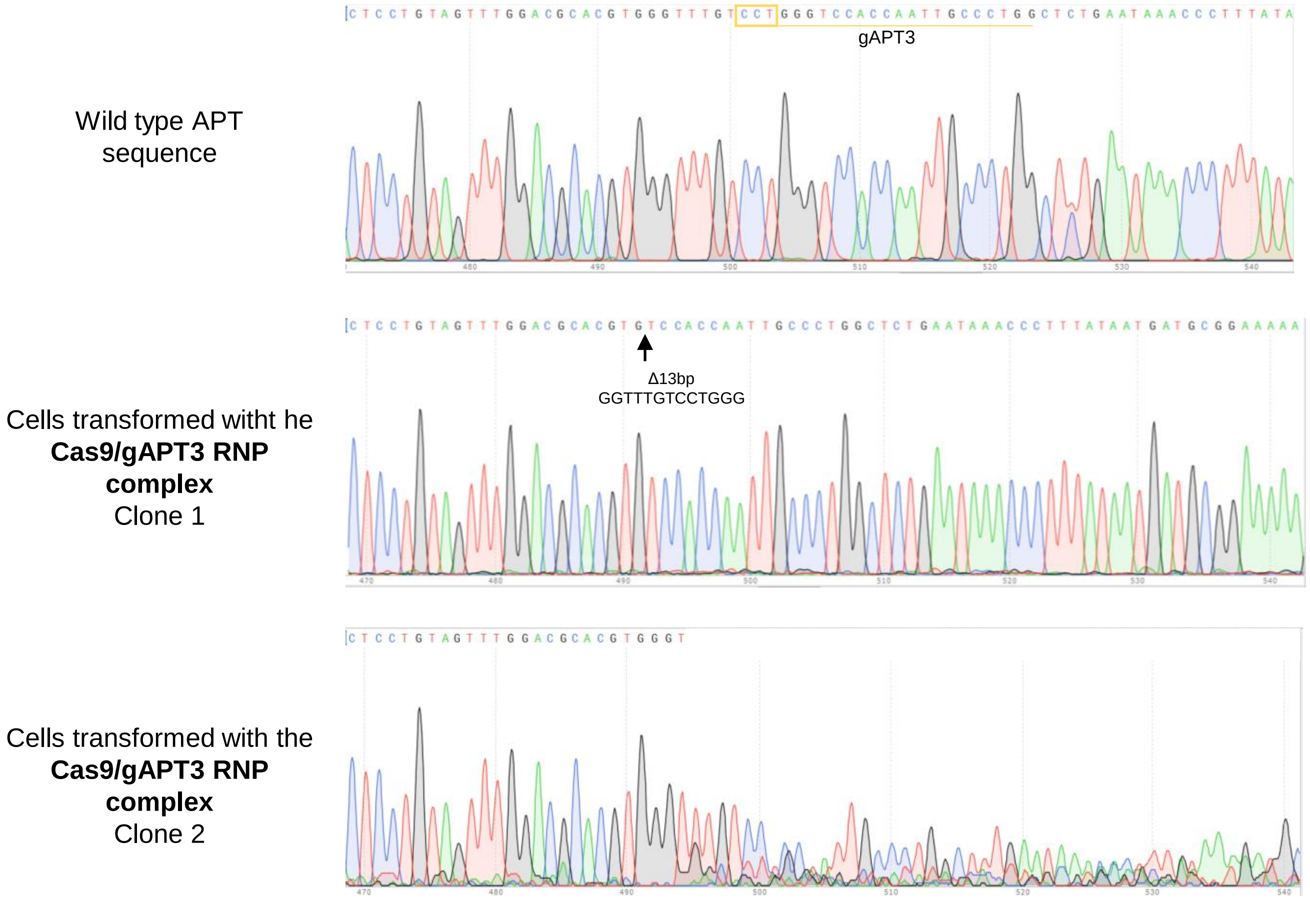

Supplementary Figure 12. Sequencing chromatograms of the gAPT3 site in wild type cells (top) and in two independent 2-FA-resistant clones obtained after transformation with the Cas9/gAPT3 RNP complex. The gRNA target site, situated on the reverse strand, is underlined and the PAM sequence (- strain: AGG) boxed.

APT genomic amplification

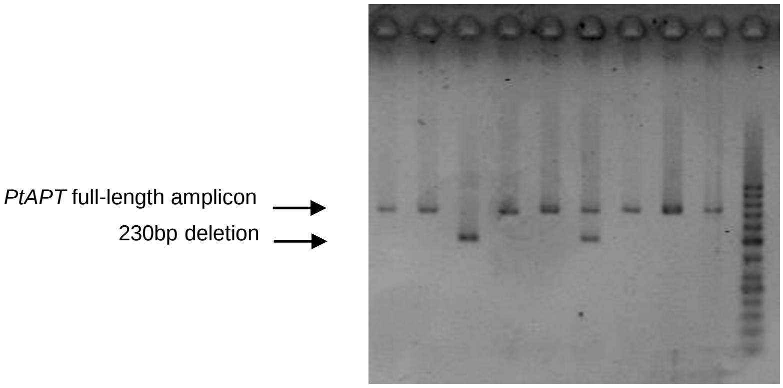

Example of mutagenic events detected at APT1 target site

WT	`TACTTTCCCTTCAAAGGCATTCCACGCTTCTACGATAT`
	`TACTTTCCCTTCAA-GGCATTCCACGCTTCTACGATAT`
	`TACTTTCCCT---AAGGCATTCCACGCTTCTACGATAT`
	TACTTTCCCTTCA**TTTCA**CGCTTCTACGATAT
	TACTTTCCCTTCAAA**A**GGCATTCCACGCTTCTACGATAT

Example of mutagenic events detected at APT3 target site

WT	`GGGTTTGTCCTGGGTCCACCA`
	GGGTTTGTCCTGGGT**T**CCACCA
	`GGGTTTGTCCTGGG-CCACCA`

Example of large deletions generated in intervening regions between two APT target sites

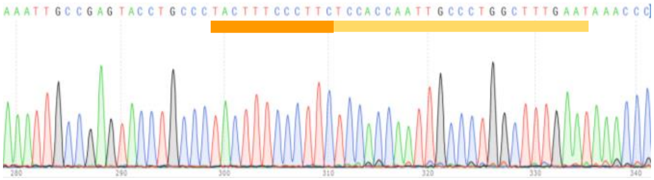

Supplementary Figure 13. Amplification of the *PtAPT* loci from 8 2-FA-resistant clones that appeared after transformation with a mixture of RNP complexes targeting *PtAPT* and *PtAureo1a*. Classical, none allele-specific PCRs were performed. Lanes 1-8: amplicon from the 2-FA^{R} clones; lane 9: amplicon from WT; lane 10: 1kb DNA ladder. Examples of mutagenic events are depicted in the below panel.

a

Experiment	Strain	Genotype
RNP UMPS/Aureo1A	UA8	[RC/WT]; [Δ115/Δ3]
	UA17	[Δ1/WT]; [Δ115/Δ4]
	UA19	[Δ319/WT]; [Δ116/ Large Ins]
RNP APT/ UMPS/ Aureo1A	AUA5	[WT/WT]; [Δ1/ WT]; [RC/ Ins]
	AUA7	[Δ319/Ins]; [Δ113/ NA]; [RC/ Δ3]
	AUA10	[RC/NA]; [Δ6/ NA]; [Δ2/ NA]
	AUA13	[WT/WT]; [Δ1/ I2]; [Δ11/ Δ29]
	AUA14	[WT/WT]; [WT/WT]; [Δ229/ Δ229]
Parental	NCMA	[WT/WT]; [WT/WT]]; [WT/ WT]
RNP APT	APTB1	[WT/ Δ230]
	APTB3	[+1/ Δ229]
RNP UMPS	UMPS4	[+1/Δ2]

b

F2 medium + 50µg mL^{-1} Uracil	F2 medium + 50µg mL^{-1} Uracil + 10µM 2-FA	F2 medium + 50µg mL^{-1} Uracil + 100µg mL^{-1} 5-FOA

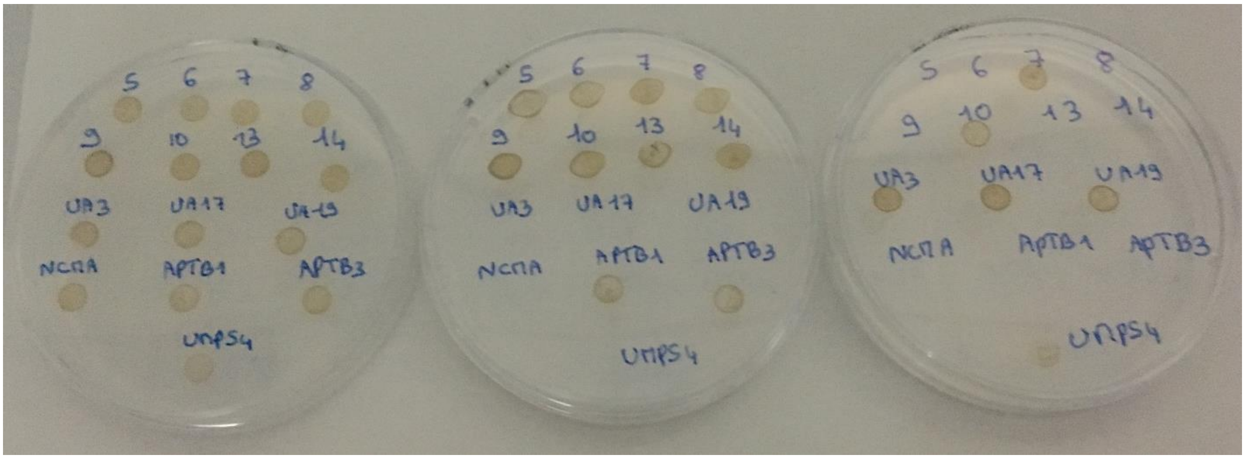

Supplementary Figure 14. Genotypic characterization and 2-FA/5-FOA resistance phenotypes of selected RNP-generated mutant strains. **(a)** Table summarizing the genotypes of the strains that were picked for additional phenotyping. The *PtUMPS* genotype for each allele is indicated in blue, the *PtAureo1a* genotype for each allele is indicated in green and the *PtAPT* genotype for each allele is indicated in orange. **(b)** Spotting assay on F/2 plates containing uracil, uracil plus 2-FA or uracil plus 5-FOA. Numbers 5 to 14 refer to strains AUA5 to AUA14. Pictures were taken after 7 days of culture.

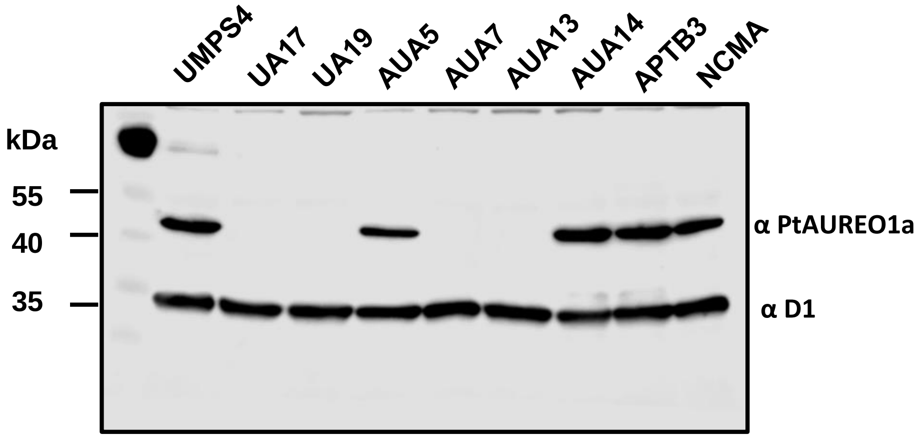

Supplementary Figure 15. Western Blot on protein samples from RNP-derived strains having no mutations at the *PtAureo1a* loci (UMPS4, AUA14, APTB3 and NCMA), of a monoallelic *PtAureo1a* mutant strain (AUA5) and of biallelic *PtAureo1a* mutant strains (UA17, UA19, AUA7 and AUA13) using a PtAureo1a antiserum (Serif et al., 2017). An antiserum against the D1 protein was used to verify that equal amounts of protein were loaded between samples.

a

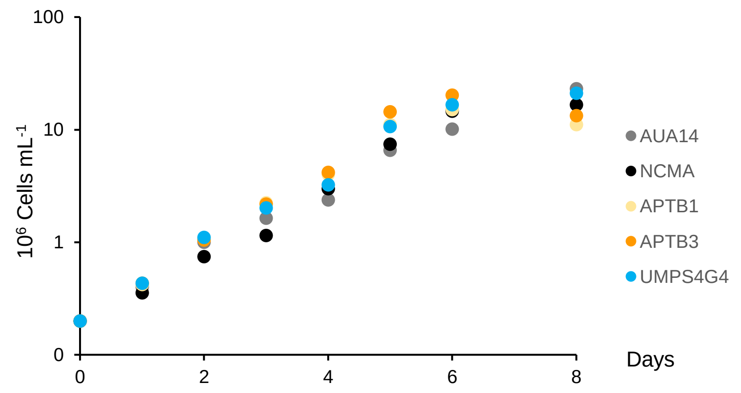

b

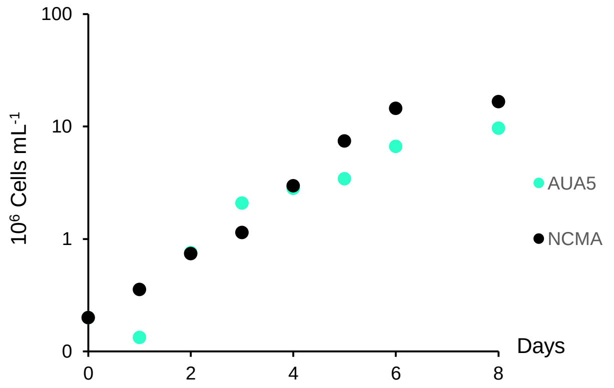

c

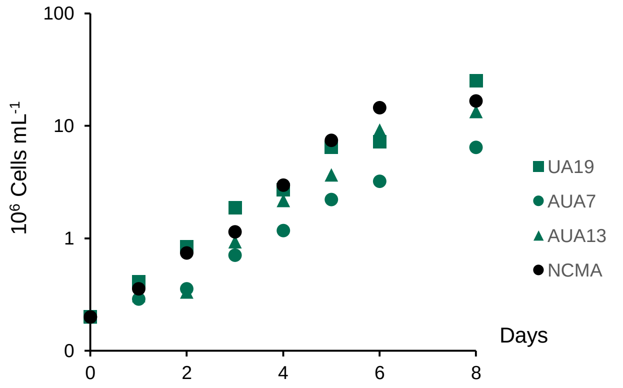

d

Experiment	Strain	Status *PtAureo 1A*	Growth rate (day^{-1})	
			Repeat 1	Repeat 2
RNP UMPS/Aureo1A	UA8	Bi-allelic	0,55	0,65
	UA17	Bi-allelic	0,64	0,69
	UA19	Bi-allelic	0,68	0,62
RNP APT/ UMPS/ Aureo1A	AUA5	Mono-allelic	0,55	0,61
	AUA7	Bi-allelic	0,44	0,52
	AUA10	Bi-allelic	0,5	0,6
	AUA13	Bi-allelic	0,65	0,64
	AUA14	WT	0,65	0,62
Parental	NCMA	WT	0,72	0,81
RNP APT	APTB1	WT	0,74	0,76
	APTB3	WT	0,82	0,81
RNP UMPS	UMPS4	WT	0,75	0,77

Supplementary Figure 16. Mutations at the *PtAureo1a* loci do not have any impact on growth. **(a to c)** Representative growth curves out of n=2 independent experiments for **(a)** various strains presenting no mutations at the *PtAureo1a* loci, **(b)** a monoallelic *PtAureo1a* mutant and **(c)** some biallelic *PtAureo1a* mutants. In **(b)** and **(c)**, NCMA was included as a control. Y-axis **(a to c)** presents cell density, expressed as million cells per mL, on a logarithmic scale. **(d)** Table summarizing the calculated exponential growth rates (day^{-1}) out of the two independent experiments.

**Condition**	**Number of 5-FOA resistant colonies**	**Number of colonies with a TM event or lack of amplification**	**Nature of the mutagenic event**
**Without guide**	0	NA	NA
**Cas9 + gUMPS1**	3	3	Clone 1: No amplification
			Clone 2: 1A Insertion
			Clone 3: Large insertion
**Cas9 + gUMPS4**	9	7	Clone 1: 1A Insertion
			Clone 2: Large deletion
			Clone 3: 1A insertion
			Clone 4: 80bp insertion
			Clone 5: Large insertion
			Clone 6: 5bp deletion
			Clone 7: Large insertion

Supplemental Table 1. Number of clones presenting TM at the *PtUMPS* loci after transformation with the Cas9- and guide RNA-encoding plasmids followed by direct selection into 5-FOA plus uracil containing medium.

Condition	Number of 5-FOA resistant colonies	Number of clones resistant in the second round of selection	Number of colonies with TM event or lack of amplification	Nature of mutagenic event
**RNP Cas9 w/o guide**	0	Not applicable	Not applicable	Not applicable
**RNP Cas9 gUMPS1**	3	1	1	No amplification
**RNP Cas9 gUMPS3**	2	1	1	No amplification
**RNP Cas9 gUMPS4**	4	4	4	▪ 2 clones +1T<br>▪ 1 clone Δ2/+1<br>▪ 1 clone Δ783/ Mosaic on the second allele

Supplemental Table 2. Number of clones presenting TM at the *PtUMPS* loci after transformation with the Cas9 protein alone or with Cas9/gRNA ribonucleoprotein complexes followed by direct selection into 5-FOA plus uracil containing medium.

Name	Sequences
TALEN UMPS	TGTCAAAACATAATACCGGAtgatgtgccgattttgttGGATGTCAAGCGCGGC GACA (+)
gUMPS1	GATGTCAAGCGCGGCGACAT**TGG** (+)
gUMPS3	CAAGCTTGTTGGCTCGGAAT**GGG** (+)
gUMPS4	TGTAGATGACGTAATCACAG**CGG** (+)
gAureo1a2	ATGAAGACCGTGTCTCCGTC**AGG** (-)
gAureo1a3	TACCAACCCGTCCAGCAAGG**TGG** (+)
gAPT1	AAGCGTGGAATGCCTTTGAA**GGG** (-)
gAPT3	CCAGGGCAATTGGTGGACCC**AGG** (-)

Supplemental Table 3. Sequences targeted by the TALEN or the CRISPR/Cas9 systems used in this study.

Biolistic experiment number	Targeted genes	Selection conditions	Number of colonies mutated on the marker	Number of colonies mutated on at least one of the other target
#1	UMPS + Aureo1A	5-FOA	17	17/17 (100%)
#2	APT + Aureo1A	2-FA	29	19/29 (66%)
#3	APT + UMPS + Aureo1A	2-FA	13	10/13 (77%)

Supplemental Table 4. Generation of multiple knock-out strains out of three independent multiplexing experiments.

Use	Locus	Forward	Reverse
PCR primers used to characterize strains from DNA transformation and for RNP *in vitro* experiments	UMPS	GAAGAAAATCGCTGTGACGC*	GTCCGTAGCTTTGCTGATACC*
	Aureo1a	GTCGCTGCTGTAACAGACA*	CTGCTGGAGGGATTCCAA*
	APT	TCCTCTGTTCGACTGCTCGT*	ACCAAACTAACGGCCGACC*
PCR Primers used for Deep sequencing experiment	UMPS	AACATTGCCTTACACGGCCT	TTGGATCCAGGATTTGACGTT
Allele-specific primers used for genetic characterization of clones from RNP experiments	UMPS Allele1	TTCGAGGCGTTAGGCGA**C***	GAGCCGAATTTGAGAACACCT**A**
	UMPS Allele2	TTCGAGGCGTTAGGCGA**T***	GAGCCGAATTTGAGAACACCT**T**
	Aureo1a Allele1	CCATGACCGACAACAACAA**A***	ATGAGGGTATCGGCCTTTTC**T**
	Aureo1a Allele2	CCATGACCGACAACAACAA**G***	ATGAGGGTATCGGCCTTTTC**G**
	APT Allele1	CGACATACCCGGAGGTTAGT**T***	CGCATCATTATAAAGGGTTTATTCA**A**
	APT Allele2	CGACATACCCGGAGGTTAGT**G***	CGCATCATTATAAAGGGTTTATTCA**G**

Supplemental Table 5. Primer sequence information. The information marked with an asterisk (*) corresponds to primers used to sequence the products.
